# Early Detection for Colorectal Cancer: ASCO Resource-Stratified Guideline

**DOI:** 10.1200/JGO.18.00213

**Published:** 2019-02-25

**Authors:** Gilberto Lopes, Mariana C. Stern, Sarah Temin, Ala I. Sharara, Andres Cervantes, Ainhoa Costas-Chavarri, Rena Engineer, Chisato Hamashima, Gwo Fuang Ho, Fidel David Huitzil, Mona Malekzadeh Moghani, Govind Nandakumar, Manish A. Shah, Catherine Teh, Sara E. Vázquez Manjarrez, Azmina Verjee, Rhonda Yantiss, Marcia Cruz Correa

**Affiliations:** ^1^ University of Miami, Sylvester Comprehensive Cancer Center, Miami, FL; ^2^ Keck School of Medicine of University of Southern California, Los Angeles, CA; ^3^ American Society of Clinical Oncology, Alexandria, VA; ^4^ American University of Beirut, Beirut, Lebanon; ^5^ Hospital Clinico Universitario, Valencia, Spain; ^6^ Rwanda Military Hospital, Kigali, Rwanda; ^7^ Tata Memorial Centre, Mumbai, India; ^8^ National Cancer Center, Tokyo, Japan; ^9^ University of Malaya, Kuala Lumpur, Malaysia; ^10^ Instituto Nacional de Ciencias Médicas y Nutrición Salvador Zubirán, Mexico City, Mexico; ^11^ Shahid Beheshti University, Tehran, Iran; ^12^ Columbia Asia Hospitals, Bangalore, India, and Weill Cornell Medical College, New York, NY; ^13^ New York-Presbyterian/Weill Cornell Medical Center, New York, NY; ^14^ Makati Medical Center, Makati, Philippines; ^15^ Homerton University Hospital Foundation Trust, Bowel Disease Research Foundation, London, United Kingdom; ^16^ The University of Puerto Rico, San Juan, Puerto Rico, and MD Anderson Cancer Center, Houston, TX

## Abstract

**PURPOSE:**

To provide resource-stratified, evidence-based recommendations on the early detection of colorectal cancer in four tiers to clinicians, patients, and caregivers.

**METHODS:**

American Society of Clinical Oncology convened a multidisciplinary, multinational panel of medical oncology, surgical oncology, surgery, gastroenterology, health technology assessment, cancer epidemiology, pathology, radiology, radiation oncology, and patient advocacy experts. The Expert Panel reviewed existing guidelines and conducted a modified ADAPTE process and a formal consensus-based process with additional experts (Consensus Ratings Group) for two round(s) of formal ratings.

**RESULTS:**

Existing sets of guidelines from eight guideline developers were identified and reviewed; adapted recommendations form the evidence base. These guidelines, along with cost-effectiveness analyses, provided evidence to inform the formal consensus process, which resulted in agreement of 75% or more.

**CONCLUSION:**

In nonmaximal settings, for people who are asymptomatic, are ages 50 to 75 years, have no family history of colorectal cancer, are at average risk, and are in settings with high incidences of colorectal cancer, the following screening options are recommended: guaiac fecal occult blood test and fecal immunochemical testing (basic), flexible sigmoidoscopy (add option in limited), and colonoscopy (add option in enhanced). Optimal reflex testing strategy for persons with positive screens is as follows: endoscopy; if not available, barium enema (basic or limited). Management of polyps in enhanced is as follows: colonoscopy, polypectomy; if not suitable, then surgical resection. For workup and diagnosis of people with symptoms, physical exam with digital rectal examination, double contrast barium enema (only in basic and limited); colonoscopy; flexible sigmoidoscopy with biopsy (if contraindication to latter) or computed tomography colonography if contraindications to two endoscopies (enhanced only).

## INTRODUCTION

The purpose of this guideline is to provide expert guidance on the early detection of colorectal cancer to clinicians, public health leaders, and policymakers in all resource settings. The target population is people at average risk of colorectal cancer. For one clinical question, the target population is persons with symptoms suspicious of colorectal cancer but not (yet) diagnosed. Asymptomatic people with elevated hereditary risk of colorectal cancer should refer to Hereditary Colorectal Cancer Syndromes: ASCO Endorsement of the Familial Risk–Colorectal Cancer: European Society for Medical Oncology (ESMO) Clinical Practice Guidelines.^1^ The guideline also does not address persons at elevated risk due to nonhereditary reasons, such as inflammatory bowel disease. This ASCO guideline focuses on the role of the early detection of colorectal cancer and the management of any polyps found during colorectal cancer screening among those at average risk as well as the workup and diagnosis of colorectal cancer.

Historically, some of the highest incidence rates have been in so-called more-developed regions, including North America, Australia, New Zealand, Western Europe, Japan, and South Korea.^2,3^ However, approximately 45% of incident colorectal cancers in men and women (in 2012) occurred in less-developed regions (the term often overlaps with terms "low-" and "middle-income countries" around the world, and represented 9% to 10% of cancers among people in those regions.^2^ In 2012, 52% of deaths resulting from colorectal cancer occurred in these less-developed regions.

These numbers are increasing around the world (eg, increases in occurrences in some Eastern European countries and Japan, increases in deaths in some South American countries and East Europe). Different regions of the world, both among and within countries, differ with respect to access to early detection (few countries outside of high-income countries have mass or even opportunistic screening). As a result of these disparities, the American Society of Clinical Oncology (ASCO) Resource-Stratified Guidelines Advisory Group chose colorectal cancer as a priority topic for guideline development. This guideline accompanies another ASCO Resource-Stratified Guideline on the treatment and follow-up of patients with early-stage colorectal cancer. A third guideline on the treatment and follow-up of patients with late-stage colorectal cancer in resource-constrained settings is in development as of this writing.

**THE BOTTOM LINE**

**Early Detection for Colorectal Cancer: ASCO Resource-Stratified Guideline**

**Guideline Question**

(1) What are the optimal strategies for population-level early detection of colorectal cancer in high-incidence and resource-constrained settings? (2) What is the optimal reflex testing strategy for people with positive screening results? (3) What is the optimal strategy for people with premalignant polyps or other abnormal screening results? (4) What are the optimal methods of diagnosis for patients with signs and symptoms of early colorectal cancer?

**Target Population**

For people who are asymptomatic, are ages 50 to 75 years, with no family history of colorectal cancer, are at average risk, and are in settings with high incidence of colorectal cancer or for adult patients with symptoms suspicious for colorectal cancer

**Target Audience**

Patients, caregivers, gastroenterologists, surgeons, medical oncologists, radiation oncologists, primary care providers, health planners, policy makers

**Methods**

A multinational, multidisciplinary Expert Panel was convened to develop clinical practice guideline recommendations based on a systematic review of existing guidelines and a formal consensus process.

**Key Recommendation Summaries**

**Screening: asymptomatic, average-risk population, high-incidence areas, age 50 to 75 years**

- Basic setting options include the following: should receive highly sensitive guaiac fecal occult blood test (gFOBT) every 1 (preferred) to 2 years if resources are available (Evidence quality: high; Strength of recommendation: strong) or may receive fecal immunochemical testing (FIT), if available, every 1 (preferred) to 2 years (Evidence quality: intermediate; Strength of recommendation: moderate)
- Limited setting options include the following: should receive highly sensitive gFOBT annually (Evidence quality: high; Strength of recommendation: strong) or may receive FIT annually (Evidence quality: intermediate; Strength of recommendation: moderate) **or should receive flexible sigmoidoscopy every 5 years (Evidence quality: high; Strength of recommendation: strong) or may receive flexible sigmoidoscopy every 10 years plus FIT (or, if FIT not available, then FOBT) every year (Evidence quality: intermediate; Strength of recommendation: strong)**
- Enhanced setting options include the following: should receive highly sensitive gFOBT annually (Evidence quality: high; Strength of recommendation: strong) or may receive FIT annually (Evidence quality: intermediate; Strength of recommendation: moderate) or should receive flexible sigmoidoscopy every 5 years (Evidence quality: high; Strength of recommendation: strong) **or may receive flexible sigmoidoscopy every 10 years plus FIT every year (Evidence quality: intermediate; Strength of recommendation: strong) or may receive colonoscopy every 10 years (Evidence quality: low; Strength of recommendation: weak)**
- Maximal setting options include the following: should receive highly sensitive gFOBT annually (Evidence quality: high; Strength of recommendation: strong) or may receive FIT annually (Evidence quality: intermediate; Strength of recommendation: moderate) or should receive flexible sigmoidoscopy every 5 years (Evidence quality: high; Strength of recommendation: strong) or may receive Flexible sigmoidoscopy every 10 years plus FIT every year (Evidence quality: intermediate; Strength of recommendation: strong) or may receive colonoscopy every 10 years (Evidence quality: low; Strength of recommendation: weak) **or may receive computed tomography (CT) colonography (Evidence quality: low; Strength of recommendation: weak) or may receive FIT DNA (Evidence quality: low; Strength of recommendation: weak)**

Note: Bold text indicates intervention added at the specific resource level.

**Reflex testing:**

If patients have a positive result from colorectal cancer screening:

- Basic/limited: then clinicians should refer patients to colonoscopy (first choice) or sigmoidoscopy (second choice and the only option for basic) if available; however, since endoscopy is not available in most basic settings, clinicians should perform or refer patients to reflex testing with double contrast barium enema (If a patient’s barium enema results are positive, refer to colonoscopy, if available; otherwise, refer the patient to surgery; Type: informal consensus; Evidence quality: insufficient; Strength of recommendation: strong).
- Enhanced/maximal: If patients have a positive result from a noncolonoscopy colorectal cancer screening, then clinicians should perform or refer patients to a colonoscopy. (Type: informal consensus; Evidence quality: insufficient; Strength of recommendation: strong).

**For people with positive premalignant polyps or other abnormal screening results—pedunculated, enhanced/maximal, overarching—refer patients to endoscopy if available and feasible; otherwise, refer to surgery.**

- Colonoscopy should be performed always with therapeutic intent (Evidence quality: insufficient; Strength of recommendation: strong), and it should be performed by endoscopist with training in polypectomy (Evidence quality: low; Strength of recommendation: strong).
- Lesions should be removed with polypectomy (Evidence quality: intermediate; Strength of recommendation: strong).
- Patients with large premalignant lesions not suitable for endoscopic resection should be referred for surgical resection (Evidence quality: insufficient; Strength of recommendation: strong).
- If lesion cannot be removed or if large lesion has a high likelihood of malignancy (Type: informal consensus), mucosal tattooing may be performed (Evidence quality: insufficient; Strength of recommendation: weak).
- Removed lesions should be retrieved for histologic exam; confirm negative borders of resection (Evidence quality: insufficient; Strength of recommendation: strong).
- Referral to surgery: Only patients with lesions that cannot be removed endoscopically should be referred to surgery (Evidence quality: insufficient; Strength of recommendation: strong).

**For nonpedunculated, enhanced/maximal:**

- Colonoscopy should be performed by endoscopists with training in large complex polyps (Evidence quality: low; Strength of recommendation: weak) always with therapeutic intent; endoscopic resection is first-line therapy for large nonpedunculated colorectal polyps with no suspicion of malignancy (Intent, Evidence quality: insufficient; Strength of recommendation: strong; Resection, Evidence quality: intermediate; Strength of recommendation: strong).
- Lesions should be removed with polypectomy; removal of lesions is dependent on the low likelihood of malignancy (Evidence quality: intermediate, Strength of recommendation: strong).
- Endoscopic assessment of lesion using enhanced endoscopy methods (if available, may include chromoendoscopy); clinicians should follow the BSGACGB guideline (Evidence quality: insufficient; Strength of recommendation: strong).
- If lesion cannot be removed (in BSGACGB guideline) or if large lesion has a high likelihood of malignancy, mucosal tattooing should be performed. For patients with polyps that are completely removed, clinicians may perform tattooing for surveillance purposes (Evidence quality: insufficient; Strength of recommendation: strong).
- Removed lesions should be retrieved for histologic exam; confirm negative borders of resection (Evidence quality: insufficient; Strength of recommendation: strong).
- Referral to surgery: Only patients with lesions that cannot be removed endoscopically should be referred to surgery (Evidence quality: insufficient; Strength of recommendation: strong).

**Optimal strategy for workup/diagnosis for those with symptoms:**

- Basic/limited: physical exam with digital rectal examination (DRE; Type: informal consensus; Evidence quality: insufficient), Double contrast barium enema (Type: informal consensus; Evidence quality: insufficient); colonoscopy with biopsy if no contraindications and available. If contraindications to colonoscopy, then flexible sigmoidoscopy with biopsy barium enema (Evidence quality: low; Strength of recommendation: weak)
- Limited: see basic/limited recommendations. Also, if incomplete colonoscopy, barium enema (Type: informal consensus; Evidence quality: insufficient; Strength of recommendation: strong)
- Enhanced: colonoscopy with biopsy if no contraindications;if contraindications to colonoscopy, flexible sigmoidoscopy with biopsy, if no contraindication, **with full visualization of the colon (barium enema or CT colonography; Evidence quality: low; Strength of recommendation: weak); CT colonography if contraindications to both of the endoscopy options or double contrast enhanced barium enema (Evidence quality: high, Strength of recommendation: moderate)**
- If incomplete colonoscopy, a double contrast enhanced barium enema or CT colonography (for colonography, if the local radiology service can demonstrate competency in this technique) (Evidence quality: intermediate; Strength of recommendation: strong)
- Maximal: physical exam with DRE (Type: informal consensus; Evidence quality: insufficient); colonoscopy with biopsy if no contraindications and available; flexible sigmoidoscopy with biopsy, if no contraindication, with full visualization of the colon (barium enema or CT colonography; Evidence quality: low; Strength of recommendation: weak); CT colonography if contraindications to both of the endoscopy options or double contrast enhanced barium enema (Evidence quality: high; Strength of recommendation: moderate)
- **Repeat colonoscopy**: If not feasible, the next tier would be one of the two following options: CT colonography (if the local radiology service can demonstrate competency in this technique) or barium enema (Evidence quality: intermediate, Strength of recommendation: strong).

Note: Bold text indicates intervention added at the specific resource level

***Additional Resources*:**

More information, including a Data Supplement with additional evidence tables, a Methodology Supplement with information about evidence quality and strength of recommendations, slide sets, and clinical tools and resources, is available at www.asco.org/resource-stratified-guidelines. Patient information is available at www.cancer.net.

**ASCO believes that cancer clinical trials are vital to inform medical decisions and improve cancer care and that all patients should have the opportunity to participate.**

ASCO has established a process for resource-stratified guidelines, which includes mixed methods of guideline development, adaptation of the clinical practice guidelines of other organizations, and formal expert consensus. This article summarizes the results of that process and presents the practice resource-stratified recommendations, which are based in part on expert consensus and adaptation from existing guidelines (see Results and Appendix Table A1).

In developing resource-stratified guidelines, ASCO has adopted its framework from the four-tier resource setting approach (basic, limited, enhanced, maximal; Table 1) developed by Breast Health Global Initiative and modifications to that framework based on the *Disease Control Priorities 3*.^4,5^ The framework emphasizes that variations occur not only between but within countries with disparities, for example, between rural and urban areas or between areas with basic primary care and more-resourced medical care not available in the local area but rather available further away. Table 2 intends to identify the setting to guideline users. ASCO uses an evidence-based approach to inform guideline recommendations.

**TABLE 1:**
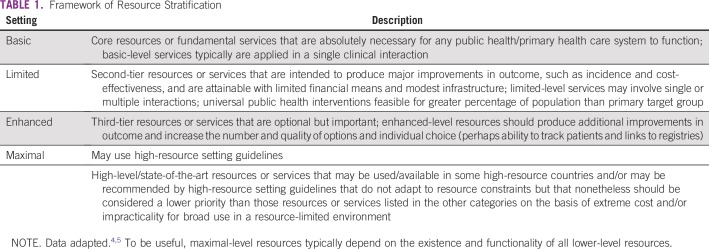
Framework of Resource Stratification

**TABLE 2:**
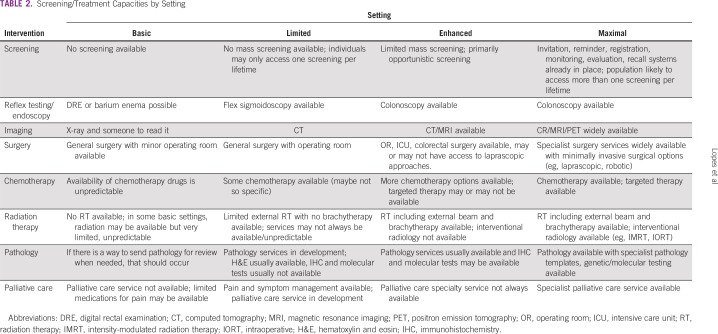
Screening/Treatment Capacities by Setting

## GUIDELINE QUESTION

This clinical practice guideline addresses the following overarching clinical questions: (1) What are the optimal strategies for population-level early detection of colorectal cancer in high-incidence and resource-constrained settings? (2) What is the optimal reflex testing strategy for people with positive screening results? (3) What is the optimal strategy for people with premalignant polyps or other abnormal screening results? (4) What are the optimal methods of diagnosis for patients with signs and symptoms of early colorectal cancer?

## METHODS

These recommendations were developed by an Expert Panel with multinational and multidisciplinary representation that included a patient representative and an ASCO guidelines staff member with health research methodology expertise (Appendix Table A2). The Expert Panel met via teleconference and in person and corresponded through e-mail. Based upon the consideration of the evidence, the authors were asked to contribute to the development of the guideline, provide critical review, and finalize the guideline recommendations. Members of the Expert Panel were responsible for reviewing and approving the penultimate version of the guideline, which was then circulated for external review and submitted to a peer-reviewed journal for editorial review and consideration for publication. This guideline was partially informed by the ASCO modified Delphi Formal Expert Consensus methodology, during which the Expert Panel was supplemented by additional experts recruited to rate their agreement with the drafted recommendations. The entire membership of experts is referred to as the Consensus Panel (the Data Supplement provides a list of members). All ASCO guidelines are ultimately reviewed and approved by the Expert Panel and the ASCO Clinical Practice Guidelines Committee (CPGC) before publication.

This guideline adaptation was also informed by the ADAPTE methodology^6^ and consensus processes used together as an alternative to de novo development for this guideline. Adaptation of guidelines is considered by ASCO in selected circumstances when one or more quality guidelines from other organizations already exist on the same topic. The objective of the ADAPTE process is to take advantage of existing guidelines to enhance efficient production, reduce duplication, and promote the local uptake of quality guideline recommendations.

ASCO adaptation and formal consensus processes begin with a literature search to identify candidate guidelines for adaptation. Adapted guideline manuscripts are reviewed and approved by the CPGC. The review includes two parts: methodologic review and content review.^7^ The methodologic review was completed by ASCO senior guideline staff (Methodology Supplement). The content review was completed by the ASCO Expert Panel.

The guideline recommendations were crafted, in part, using the Guidelines Into Decision Support (GLIDES) methodology.^8^ Detailed information about the methods used to develop this guideline is available in the Methodology Supplement and Data Supplement at www.asco.org/resource-stratified-guidelines.

The ASCO Expert Panel and guidelines staff will work with co-chairs to keep abreast of any substantive updates to the guideline. On the basis of formal review of the emerging literature, ASCO will determine the need to update.

This is the most recent information as of the publication date. For updates, for the most recent information, and to submit new evidence, please visit www.asco.org/resource-stratified-guidelines. All funding for the administration of the project was provided by ASCO.

### Guideline Disclaimer

The clinical practice guidelines and other guidance published herein are provided by the American Society of Clinical Oncology, Inc. (“ASCO”) to assist providers in clinical decision making. The information therein should not be relied upon as being complete or accurate, nor should it be considered as inclusive of all proper treatments or methods of care or as a statement of the standard of care. With the rapid development of scientific knowledge, new evidence may emerge between the time information is developed and when it is published or read. The information is not continually updated and may not reflect the most recent evidence. The information addresses only the topics specifically identified therein and is not applicable to other interventions, diseases, or stages of diseases. This information does not mandate any particular course of medical care. Further, the information is not intended to substitute for the independent professional judgment of the treating provider, as the information does not account for individual variation among patients. Recommendations reflect high, moderate or low confidence that the recommendation reflects the net effect of a given course of action. The use of words like “must,” “must not,” “should,” and “should not” indicate that a course of action is recommended or not recommended for either most or many patients, but there is latitude for the treating physician to select other courses of action in individual cases. In all cases, the selected course of action should be considered by the treating provider in the context of treating the individual patient. Use of the information is voluntary. ASCO provides this information on an “as is” basis, and makes no warranty, express or implied, regarding the information. ASCO specifically disclaims any warranties of merchantability or fitness for a particular use or purpose. ASCO assumes no responsibility for any injury or damage to persons or property arising out of or related to any use of this information or for any errors or omissions.

### Guideline and Conflict of Interest

The Expert Panel was assembled in accordance with the ASCO Conflict of Interest Policy Implementation for Clinical Practice Guidelines (“Policy,” found at http://www.asco.org/rwc). All members of the Expert Panel completed the ASCO disclosure form, which requires disclosure of financial and other interests, including relationships with commercial entities that are reasonably likely to experience direct regulatory or commercial impact as a result of promulgation of the guideline. Categories for disclosure include employment; leadership; stock or other ownership; honoraria, consulting or advisory role; speaker's bureau; research funding; patents, royalties, other intellectual property; expert testimony; travel, accommodations, expenses; and other relationships. In accordance with the Policy, the majority of the members of the Expert Panel did not disclose any relationships constituting a conflict under the Policy.

## RESULTS

### Literature Search

As part of the systematic literature review, PubMed, Cochrane Systematic Review, and National Guideline Clearinghouse databases were searched for guidelines, systematic reviews, and meta-analyses published between 2007 and September 2017. The Panel used literature searches (for primary literature, 2007 to 2017 [September]; for guidelines, 2012 to 2017 [June]), in PubMed for existing guidelines (searched National Guidelines Clearinghouse [NGC] 2017), a cost-effectiveness analysis registry, and expert consensus publications, some literature suggested by the Panel, and clinical experience as guides. Inclusion criteria identified publications that were (1) on the early detection of colorectal cancer and, if guidelines, were (2) developed by multidisciplinary content experts as part of a recognized organizational effort and (3) published between 2012 and 2017. Searches for cost-effectiveness analyses were also conducted. Articles were excluded from the systematic review if they were (1) meeting abstracts or (2) books, editorials, commentaries, letters, news articles, case reports, or narrative reviews.

A total of 39 guidelines were found in the literature search, and their currency, content, and methodology were reviewed. On the basis of content and methodology reviews, the Expert Panel chose guidelines from eight reputable public health authorities/guideline developers; Appendix Table A1 lists links to the guidelines.

This ASCO guideline reinforces selected recommendations offered in guidelines from the United States Preventive Services Task Force (USPSTF),^9^ Canadian Task Force on Preventive Health Care (CTFPHC),^10^ Cancer Care Ontario (CCO),^11^ European Commission,^12,13^ British Society of Gastroenterology/Association of Coloproctologists of Great Britain and Ireland (BSGACGB),^14^ National Guideline Alliance/National Institute for Health and Care Excellence (NICE) guidelines,^15^ and Appropriateness Criteria from the American College of Radiology (ACR),^16^ as well as an ASCO guideline endorsement (Lynch syndrome),^1^ and it acknowledges the effort put forth by the authors and aforementioned societies to produce evidence-based and/or consensus-based guidelines informing practitioners and institutions who provide early detection and diagnosis of colorectal cancer. The identified guidelines were published between 2012 and 2017. The Data Supplement includes an overview of these guidelines, including information on the clinical questions, target populations, development methodology, and key evidence.

Some US guidelines (eg, American Cancer Society [ACS], US Multi-Society Task Force [USMSTF]) have recommended screening at age 45 years. ACS makes what they called “a qualified recommendation” as opposed to the strong recommendation given for 50 years or older. This is not based on new trials but rather results from modeling of USPSTF data.^17^ The USMSTF made a weak recommendation for screening starting at age 45 years for African Americans with “very-low-quality evidence.”^18^ However, the ACS and USMSTF guidelines do not agree with other guidelines, and consensus among experts about starting earlier is not yet reached.

The ACS recommendation is a qualified recommendation. Since this ASCO guideline is based on the systematic review-based guidelines, including systematic reviews conducted for USPSTF, it affirms the USPSTF and CTFPHC recommendations.

## GUIDELINES ON EARLY DETECTION

### Clinical Questions and Target Population of Guidelines Being Adapted by ASCO

#### Note on methods.

ASCO considered quality guidelines that either met the NGC 2013 criteria as assessed by NGC or met ASCO criteria for Appraisal of Guidelines for Research and Evaluation II (AGREE II) methodologic review.

The maximal resource-level setting guidelines adapted in part by ASCO are listed in Table A1. For screening, the Expert Panel used the following guidelines as the evidence base: The USPSTF guideline, based on a Kaiser Permanente research affiliates evidence-based practice center systematic review, pertains to population screening in the high-incidence United States, with a target population of adults age 50 years or older at average risk of colorectal cancer and without a family history.^9^ The primary clinical questions concerned effectiveness, test performance characteristics, and adverse events (including in subpopulation). The systematic review–based CTFPHC guideline focuses on population screening in high-incidence Canada, with a target population of adults age 50 years or older at average risk.^10^ The primary clinical questions concerned benefits, test properties, and adverse events. Chapter 1 of the European Commission guideline (the audience is primarily in high-incidence settings) concerns the same population, a target population of the general population at an average risk and age 50 years or older. The systematic review included multiple clinical questions (n = 23) that generally pertained to effectiveness and adverse events; they are available in Appendix 1 of the European Commission guideline.^13^ This guideline also refers to the ASCO endorsement of an ESMO guideline on early detection of colorectal cancer for those at high risk by virtue of familial colorectal cancer.^1^

For the management of polyps, the Expert Panel relied upon the BSGACGB guideline. The target population of the BSGACGB guideline, based on a systematic review, is adults in the United Kingdom with large nonpedunculated colorectal polyps. The clinical questions that pertained to management options, choosing among them, and post-management follow-up were considered for this ASCO guideline.^14^ Chapter 8 of the European guideline also informed the management of polyps recommendations.^13^

For the staging/diagnosis of colon cancer, the Expert Panel used selected NICE and CCO guidelines. The NICE guideline covers the diagnosis and staging of adults with colorectal cancer, their families, and other caregivers. It is based on a systematic review, and the clinical questions of primary interest were about the effectiveness of diagnosis and staging interventions/techniques and the treatment of patients with symptoms and emergency presentation.^15^ The target population of the CCO guideline is adults with nonemergency symptoms in primary care in the high-incidence country of Canada, and the clinical question on diagnostic accuracy of tests for patients with symptoms was considered for this ASCO guideline.^11^

The ACR Appropriateness Criteria is based on systematic processes, and the target population patients with colorectal cancer. The clinical questions are not overtly stated.^16^

### Summary of Guidelines Being Adapted by ASCO: Development Methodology and Key Evidence

The USPSTF methods included systematic and nonsystematic reviews of published and gray literature and critical appraisal of the evidence with Grading of Recommendations Assessment, Development, and Evaluation (GRADE). This guideline met the 2013 NGC criteria. The evidence underlying the recommendations came from randomized clinical trials (RCTs) or observational literature, depending on the clinical (key) question.

The CTFPHC methods included a systematic review of RCTs (for benefits) and RCTs, cohort (with a comparison), and case-control (for test properties) studies. The CTFPHC used GRADE and also met the 2013 NGC criteria. The European Commission guideline was rated 71% on the AGREE II assessment by ASCO (Methodology Supplement). The literature review used systematic reviews; population and observational studies, including uncontrolled case series; and RCTs, if available.

The BSGACGB guideline was based on a systematic review using established methods, including Scottish Intercollegiate Guidelines Network and GRADE, to review randomized and observational data and received a 69% rating on the AGREE II.

The NICE guideline met the 2013 NGC criteria. The key evidence included systematic reviews, meta-analyses, RCTs, and case series. The developers noted that there were limited high-quality studies on staging disease in patients with colon cancer. NICE used GRADE methodology. The CCO guideline was based on a systematic review and met the ASCO AGREE II threshold. The evidence included primarily observational data and limited high-quality data.

The ACR Appropriateness Criteria met the NGC criteria, and its methods were based on RAND/UCLA Appropriateness Methods. ACR uses its own scheme for rating the quality of included studies and the strength of recommendations. Key evidence included diagnostic and therapeutic references of varying quality and some meta-analyses.

## OUTCOMES

The outcomes/end points in most studies reviewed by the adapted guidelines included the following: for Clinical question 1, Screening: mortality, test performance characteristics, diagnostic accuracy, and safety; for Clinical question 2, Management of polyps: recurrence, completeness of excision, safety, survival; for Clinical question 3, Diagnosis and staging: sensitivity, specificity, disease-free survival, safety (for the NICE guideline, cost-effectiveness). For the primary care guideline from CCO, the primary outcomes were mostly positive predictive value and other diagnostic accuracy end points.

## RESULTS OF ASCO METHODOLOGIC REVIEW

The methodologic review of the guidelines was completed by two ASCO guideline staff members using the Rigor of Development subscale of the AGREE II instrument. The score for the Rigor of Development domain is calculated by summing the scores across individual items in the domain and standardizing the total score as a proportion of the maximum possible score. Detailed results of the scoring and the AGREE II assessment process for this guideline are available in the Methodology Supplement.

## RECOMMENDATIONS

The recommendations were developed by a multinational, multidisciplinary group of experts using evidence from existing guidelines and clinical experience as a guide. The ASCO Expert Panel underscores that health care practitioners who implement the recommendations presented in this guideline should first identify the available resources in their local and referral facilities and endeavor to provide the highest level of care possible with those resources.

### Screening Recommendations

These recommendations (Table 3) were adapted from the following guidelines: USPSTF, CTFPHC, and Chapter 1 of the European Commission guideline.^9,10,13^

**TABLE 3:**
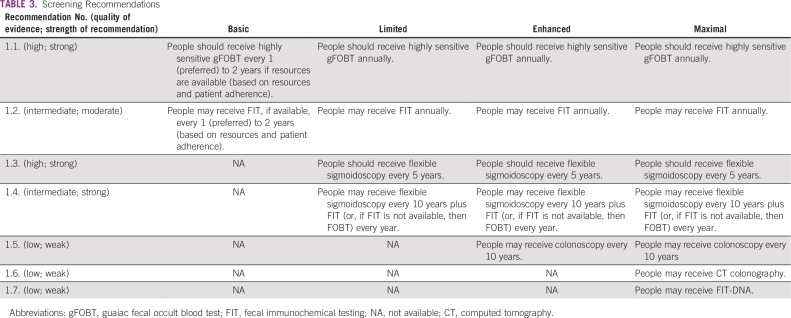
Screening Recommendations

#### Clinical question 1.

What are the optimal strategies for population-level early detection of colorectal cancer in high-incidence and resource-constrained settings?

##### General recommendations for screening.

Screening for colorectal cancer is recommended for asymptomatic people, ages 50 to 75 years, with an average risk of cancer, in settings where colorectal cancer incidence is high and/or mortality is high and/or the proportion of individuals diagnosed with advanced-stage disease is high. The ages are based on the age ranges of trial participants and span the recommended age range in the majority of systematic review-based guidelines. Although this guideline recommends screening by using certain chronologic age ranges, chronologic age can be insufficient for decision making, and clinicians may use functional age. The Expert Panel would like to emphasize that life expectancy and underlying health status are important to assess and consider. The basic rationale for cancer screening is to prevent the development of malignancy during the 10 to 15 years after an instance of screening (and to diagnose cancer earlier; ie, stage shift) and ultimately to lower cancer-specific mortality. In lower-resource settings, implementing interventions for older patients who may not benefit if they have poor health status and/or limited life expectancy is an important consideration for policy makers.

Persons with certain risk factors (eg, family history, inflammatory bowel disease, familial adenomatous polyposis, Lynch) need more frequent screening; see the ASCO Hereditary Colorectal Cancer Syndromes endorsement of an ESMO guideline. The following tests are recommended for each setting, although most of the tests recommended are not available in basic and limited settings.^1^

#### Basic setting.

*Recommendation 1.1.* People should receive highly sensitive guaiac fecal occult blood test (gFOBT), preferably every year, or alternatively every 2 years, depending on available resources and patient adherence in the population of interest (Evidence quality: high; Strength of recommendation: strong).
*Recommendation 1.2.* If available, people may receive fecal immunochemical testing (FIT) instead of gFOBT, preferably every year, or every 2 years, depending on resources and patient adherence in the population of interest (Evidence quality: intermediate; Strength of recommendation: moderate).

These recommendations reflect the presumption that colonoscopy is not available in these settings, though the Expert Panel suggests that policymakers develop colonoscopy services. The basic rationale for screening with gFOBT or FIT is cancer detection at earlier stages and prevention of death.

#### Limited setting.

In limited settings, people should receive the same screening approaches as the basic setting.

*Recommendation 1.3.* If available, they should receive flexible sigmoidoscopy every 5 years (Evidence quality: high; Strength of recommendation: strong).
*Recommendation 1.4.* People may receive flexible sigmoidoscopy every 10 years plus FIT every year, or gFOBT every year if FIT is not available (Evidence quality: intermediate; Strength of recommendation: strong).

These recommendations reflect the presumption that colonoscopy is not likely to be available in basic and limited settings.

#### Enhanced setting.

In enhanced settings, people should receive the same screening approaches as recommended for those in limited settings.

*Recommendation 1.4.* Given the more common availability of FIT, flexible sigmoidoscopy should be offered every 10 years with annual FIT testing (Evidence quality: intermediate; Strength of recommendation: strong).
*Recommendation 1.5.* An alternative to all of the limited and enhanced screening procedures is to perform colonoscopy every 10 years (more frequently for those at high risk; see Stoffel et al^1^; Evidence quality: low; Strength of recommendation: weak).

#### Maximal setting.

In maximal settings, the same procedures offered in the enhanced setting should be offered to the average-risk population, with two additional alternatives.

*Recommendation 1.6.* People may receive computed tomography (CT) colonography (Evidence quality: low; Strength of recommendation: weak).
*Recommendation 1.7.* People may receive FIT DNA (Evidence quality: low; Strength of recommendation: weak).

#### Source guidelines and discussion.

The recommended age for screening was adopted from the USPSTF guideline.^9^ The CTFPHC guideline noted that the absolute benefits of screening are largest for those age 60 to 74 years rather than those age 50 to 59 years due to the increased incidence of colorectal cancer among those older than 60 years.^10^ The evidence for gFOBT was considered high, and the recommendation was strong, given the evidence from multiple clinical trials with up to 30 years of follow-up that showed up to 22% reduction in mortality when gFOBT is used every 2 years and up to 32% when used every year.^9^ The Expert Panel rated the recommendation for FIT instead of gFOBT as moderate strength because of the intermediate strength of the evidence due to lack of sufficient clinical trials; however, the evidence from systematic reviews of other studies concluded that FIT had similar specificity as gFOBT and potentially higher benefit due to higher sensitivity and increased participation of people in screening when FIT was used instead of gFOBT.^10^ The WHO International Agency for Research on Cancer (IARC) published an evaluation after the literature search for this guideline was completed and concluded that there was sufficient evidence for both gFOBT and FIT to reduce colorectal cancer mortality and that benefits outweigh the harms of screening with these procedures.^19^ The evidence for reducing incidence with FIT was limited. In addition, the evidence for higher-sensitivity gFOBT every 1 or 2 years was limited. That evidence was suggestive of or showed a lack of effect for regular gFOBT testing every 2 years.

The evidence for flexible sigmoidoscopy every 5 years was high due to the availability of several clinical trials (the USPSTF and CTFPH guidelines mention four) that led to a strong recommendation for limited, enhanced, and maximal settings.^10,14^ The combination of flexible sigmoidoscopy every 10 years with annual FIT is also strongly recommended in these settings but with intermediate evidence, given that fewer trials have investigated this approach. The IARC evaluation (in 2017) concluded that the evidence for flexible sigmoidoscopy was sufficient to reduce the incidence of colorectal cancer and to reduce mortality, and that the benefits that outweigh the risks of these procedures.^19^

The use of colonoscopy is conditionally recommended given the low evidence due to lack of randomized clinical trial data (trials are still ongoing). Even though indirect evidence from the benefit of colonoscopy can be obtained from flexible sigmoidoscopy trials, the benefits must be weighed against the possible harms due to adverse effects of bowel preparation, sedation, and the actual procedure. This recommendation will need to be revisited when clinical trial data are available. In contrast with this assessment, the evaluation conducted at IARC concluded that there is sufficient evidence that colonoscopy can reduce incidence and mortality and that the benefits outweigh the risks. However, the publication noted that a minority of panel members considered the evidence to be limited. In contrast with the ASCO Expert Panel approach, the evaluation at IARC included observational studies to assess the strength of the evidence for colonoscopy and considered the evidence for sigmoidoscopy as a surrogate for partial colonoscopy.

The ASCO recommendation for CT colonography is weak given the low strength of the evidence and concerns about overdiagnosis and overtreatment due to incidental extracolonic findings; therefore, CT colonography is only one of several screening options offered. Finally, there is a weak recommendation for FIT DNA (a DNA test is added to FIT) due to the low strength of evidence, which suggests lower specificity compared with FIT, and the lack of studies investigating follow-up of abnormal results with results of negative colonoscopy. In agreement with the ASCO assessment, the IARC evaluation concluded that there is limited evidence for CT colonography to reduce incidence and mortality and inadequate evidence to assess whether the benefits outweigh the risks.

### Reflex Testing After Positive Screening

The recommendations (Table 4) are based on informal expert consensus (and are validated by formal expert consensus).

**TABLE 4:**
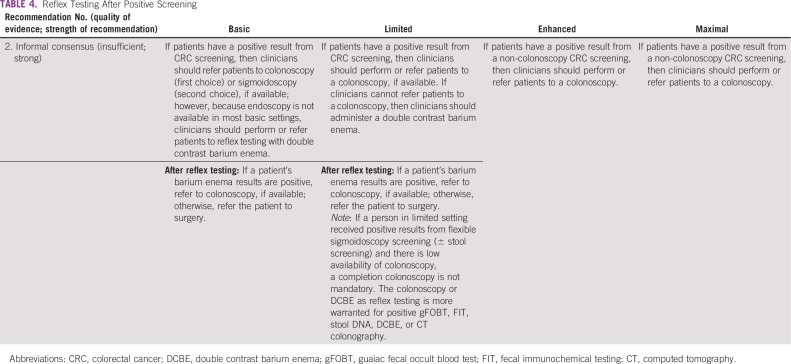
Reflex Testing After Positive Screening

#### Clinical question 2.

What is the optimal reflex testing strategy for people with positive screening results?

#### Basic setting.

*Recommendation 2.* Double contrast barium enema should follow positive stool-based screening results. If referral to colonoscopy is a possibility, then a patient with a positive stool-based test should be referred to colonoscopy. If colonoscopy referral is not available, a person who had a positive result from a barium enema should be referred to surgery to evaluate for surgical malignancy. (Type: informal consensus validated by formal consensus; Evidence quality: insufficient; Strength of recommendation: strong).

This recommendation was made with the assumption that equipment and expertise to conduct this procedure should be available in most basic settings or at nearby facilities, where a clinician can refer patients. Policymakers in basic settings should aspire to develop regional centers where clinicians could refer patients with positive screening results for colonoscopy.

#### Limited setting.

*Recommendation 2.* Patients should receive a colonoscopy, if this is available, or patients can be referred to a nearby center to receive a colonoscopy. Alternatively, patients should receive a double contrast barium enema for the same reasons outlined for the basic setting. If patients have positive results from FOBT/FIT and then have a negative barium enema result, they should continue annual FIT evaluation (Type: informal consensus; Evidence quality: insufficient; Strength of recommendation: strong).

#### Enhanced and maximal settings.

*Recommendation 2.* Patients in these settings who were screened with noncolonoscopic procedures should receive (or be referred to receive) a colonoscopy (Type: informal consensus; Evidence quality: insufficient; Strength of recommendation: strong).

### Management of Patients With Polyps

These recommendations (Tables 5 and 6) are based on the BSGACGB guidelines and Chapter 8 of the European Commission guidelines.^13,14^ All recommendations rated informal consensus were validated by formal consensus.

**TABLE 5:**
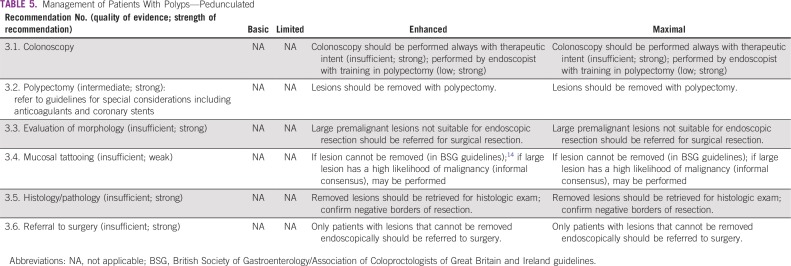
Management of Patients With Polyps—Pedunculated

**TABLE 6:**
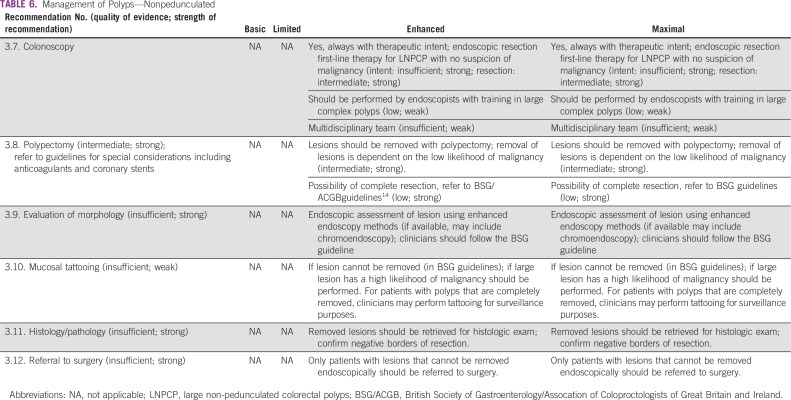
Management of Polyps—Nonpedunculated

#### Clinical question 3.

What is the optimal strategy for people with premalignant polyps or other abnormal screening results?

#### Enhanced and maximal settings.

The following resource-stratified recommendations on management of patients with colorectal polyps are based on the availability of endoscopic equipment and proficiency in polypectomy techniques that specialists such as gastroenterologists and surgeons require. In basic and limited-resource settings, endoscopic facilities, equipment for standard polypectomy, and trained endoscopists are generally unavailable or scarce. In enhanced and maximal settings, endoscopic facilities are available (including for colonoscopies), are equipped with polypectomy equipment (eg, snares/injectors/clips), and have trained endoscopists that can offer diagnosis and treatment of simple and complex polyps.

Colonoscopy should always be done with therapeutic intent; thus, the endoscopist who conducts the screening or surveillance colonoscopy should have the necessary expertise to perform polypectomies of all but the most demanding colorectal polyps. There is abundant evidence that colorectal adenomas are premalignant (Chapter 8 of the European Steele and the British Guidelines),^13,14^ and, when a clinician finds a lesion during colonoscopy that could be an adenoma, they should remove it.

Colonic lesions should only be removed by endoscopists with adequate training in polypectomy techniques. For the purposes of management, polyps may be classified according to location (colon *v* rectum), by morphology (pedunculated *v* sessile), and by size (< 5 mm, 5 to 9 mm, l0 to 19 mm, ≥ 20 mm). If patients have large colorectal polyps that are not suitable for endoscopic resection or if endoscopic excision is not possible due to lack of expertise, clinicians should refer the patients for surgical resection.

#### Source guidelines and discussion.

The recommendations for the management of patients with colorectal polyps (Tables 5 and 6) are discussed separately based on the type of morphology of the colorectal lesions—pedunculated and nonpedunculated (pedunculated polyps include stalks).

The evidence was relatively stronger for polypectomy for both pedunculated and nonpedunculated polyps than for other recommendations in this section. In general, however, the recommendations were based on observational data (eg, with populations of prospective or retrospective cohorts); Chapter 8 of the European guideline was primarily based on uncontrolled case series with some limited RCT data.^13^

##### 3a: Pedunculated.

The recommendations are adapted primarily from the European guidelines and are only for enhanced and maximal settings, as endoscopic management of polyps requires the availability of endoscopic equipment (including colonoscopies) and polypectomy accessories and proficient endoscopists. If sigmoidoscopes and other equipment for polypectomy are available in the limited setting, the guidelines would be applicable for management of pedunculated polyps in that setting, also.

*Recommendation 3.1.* Colonoscopy should be performed always with therapeutic intent (Evidence quality: insufficient; Strength of recommendation: strong) and performed by an endoscopist with training in polypectomy (Evidence quality: low; Strength of recommendation: strong).
*Recommendation 3.2.* Lesions should be removed with polypectomy (Evidence quality: intermediate; Strength of recommendation: strong).
*Recommendation 3.3.* Patients with large premalignant lesions not suitable for endoscopic resection should be referred for surgical resection (Evidence quality: insufficient; Strength of recommendation: strong).
*Recommendation 3.4.* If lesion cannot be removed (Type: evidence-based) or if large lesion has a high likelihood of malignancy (Type: informal consensus), mucosal tattooing may be performed (Evidence quality: insufficient; Strength of recommendation: weak).
*Recommendation 3.5.* Removed lesions should be retrieved for histologic exam; confirm negative borders of resection (Evidence quality: insufficient; Strength of recommendation: strong).
*Recommendation 3.6.* Only patients with lesions that cannot be removed endoscopically should be referred to surgery (Evidence quality: insufficient; Strength of recommendation: strong).

The European guidelines noted the benefits and potential harms: The benefits include reducing the risk of the development of malignancy by removing polyps that may be precursors; the potential harms are general risks of endoscopy, including perforation and bleeding.

##### 3b: Nonpedunculated.

These recommendations are based on the BSGACGB and European Commission guidelines (the former addresses solely nonpedunculated polyps).^13,14^

*Recommendation 3.7.* Colonoscopy should be performed always with therapeutic intent (Evidence quality: insufficient; Strength of recommendation: strong); endoscopic resection should be performed as first-line therapy for large nonpedunculated colorectal polyps with no suspicion of malignancy (for intent, Evidence quality: insufficient; Strength of recommendation: strong; for resection, Evidence quality: intermediate; Strength of recommendation: strong). Colonoscopy should be performed by endoscopists with training in large complex polyps (Evidence quality: low; Strength of recommendation: weak). A multidisciplinary team may perform colonoscopies (Evidence quality: insufficient, Strength of recommendation: weak).
*Recommendation 3.8.* Lesions should be removed with polypectomy; removal of lesions is dependent on the low likelihood of malignancy (Evidence quality: intermediate; Strength of recommendation: strong). For possibility of complete resection, refer to BSGACGB guidelines (Evidence quality: low; Strength of recommendation: strong).
*Recommendation 3.9.* For endoscopic assessment of lesion using enhanced endoscopy methods (if available, may include chromoendoscopy), clinicians should follow the BSG guideline (Evidence quality: insufficient; Strength of recommendation: strong).
*Recommendation 3.10.* If lesion cannot be removed (in BSGACGB guidelines) or if large lesion has a high likelihood of malignancy, mucosal tattooing should be performed. For patients with polyps that are completely removed, clinicians may perform tattooing for surveillance purposes (Evidence quality: insufficient; Strength of recommendation: weak).
*Recommendation 3.11.* Removed lesions should be retrieved for histologic exam; confirm negative borders of resection (Evidence quality: insufficient; Strength of recommendation: strong).
*Recommendation 3.12.* Only patients with lesions that cannot be removed endoscopically should be referred to surgery (Evidence quality: insufficient; Strength of recommendation: strong).

#### Source guidelines and discussion.

For clinicians who treat patients with pedunculated or nonpedunculated polyps, surgery referrals are recommended only when endoscopic removal is not possible. As noted, the evidence is insufficient; the European recommendations were based on expert opinion. BSGACGB recommends surgery for incomplete endoscopic removal of lesions (to support Recommendations 3.6 and 3.12) and when the clinician thinks there is malignancy rather than premalignancy, with moderate evidence. The benefits and risks for transanal surgical removal of very large (≥ 20 mm) sessile lesions of the rectum should be discussed as an option. Specifically, transanal endoscopic microsurgery (TEM) is the preferred method of local excision (TEM also is discussed in the ASCO resource-stratified guideline for the treatment of patients with early-stage colorectal cancer). Clinicians should attempt endoscopic mucosal resection before they make a referral to surgery; situations in which resection is not suitable include very large sessile lesions (> 40 mm in size) those in difficult locations (appendix, invading a diverticulum, and the squamocolumnar junction), or those with increased risk of malignancy (based on pit pattern and/or morphology).

For basic and limited-resource settings, management of disease in patients with positive non–colonoscopy screening tests, such as FOBTs, does not include recommendations of colonoscopy, as the recommendations specifically address the evaluation of lesions identified during colonoscopy. Although there is no evidence to inform this section for underserved communities and/or low- and middle-income countries, if polyps are found in patients in limited settings by flexible sigmoidoscopy (plus FIT or FOBT; people may receive flexible sigmoidoscopy every 10 years plus FIT or, if FIT not available, then FOBT every year) as in Recommendation 1.4, the informal consensus of the Expert Panel is to proceed with polypectomy of the lesion seen during flexible sigmoidoscopy if the polypectomy equipment is available and if there is a proficient endoscopist. If endoscopic equipment is not available, then patients with a positive screening test (eg, FOBT) should have their disease investigated by a radiologic study (eg, barium enema) and, if positive, clinicians should refer the patient to colonoscopy with polypectomy or surgery in an enhanced setting (guided by local expertise). Clinicians should complete an investigation of positive screening test results for colorectal cancer to detect colorectal adenomas, as colorectal adenomas are premalignant. When a lesion that could be an adenoma is found during colonoscopy, then clinicians should remove it. This includes positive results from flexible sigmoidoscopy that should be followed by a completion colonoscopy, if available, or barium enema, if not.

The Expert Panel also refers readers to other guidelines for special considerations, including anticoagulants and coronary stents, that are outside of the scope of this guideline, since existing guidelines exist to address this situation (eg, American Society for Gastrointestinal Endoscopy guidelines for managing anticoagulation in the setting of polypectomy^20^ or within the European guidelines, Chapter 8^13^).

### Workup and Diagnosis

These recommendations (Table 7) are based on CCO and NICE guidelines.^11,15^

**TABLE 7:**
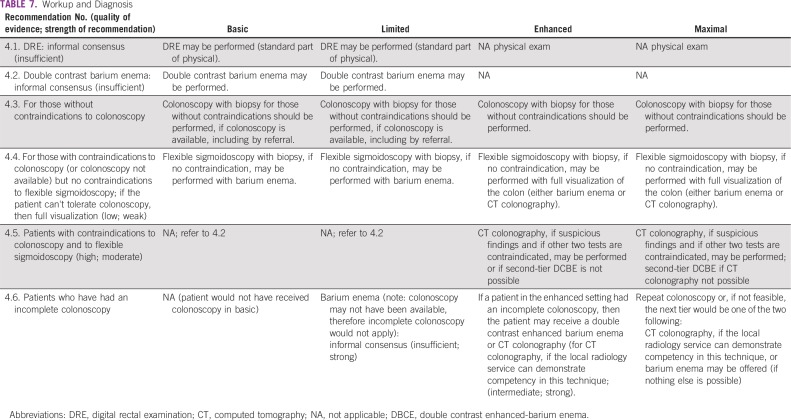
Workup and Diagnosis

#### Clinical question 4.

What is the optimal strategy for the workup and diagnosis for those with symptoms suspicious of colorectal cancer (iron deficiency anemia, bleeding, abdominal pain, and change in bowel habits)?

#### Basic and limited settings.

*Recommendation 4.1.* Digital rectal examination (DRE) may be performed (standard part of physical; Evidence quality: insufficient).
*Recommendation 4.2.* Double contrast barium enema may be performed (Evidence quality: insufficient).

#### **All settings**.

*Recommendation 4.3.* Colonoscopy with biopsy for those without contraindications should be performed, if colonoscopy is available, including by referral. In enhanced and maximal settings, colonoscopy with biopsy for those without contraindications should be performed.
*Recommendation 4.4.* For those with contraindications to colonoscopy (or colonoscopy not available) but no contraindications to flexible sigmoidoscopy, if the patient cannot tolerate colonoscopy, then provide full visualization: In basic and limited settings, flexible sigmoidoscopy with biopsy, if no contraindication, may be performed with barium enema. In enhanced and maximal settings, flexible sigmoidoscopy with biopsy, if no contraindication, may be performed with full visualization of the colon (either barium enema or CT colonography).

#### Enhanced and maximal settings.

*Recommendation 4.5.* CT colonography with biopsy may be performed if suspicious findings and if other two tests are contraindicated, or, if second-tier DCBE is not possible (Evidence quality: high; Strength of recommendation: moderate).
*Recommendation 4.6.* If a patient in the enhanced setting had an incomplete colonoscopy, then the patient may receive a double contrast enhanced barium enema or CT colonography (for CT colonography, if the local radiology service can demonstrate competency in this technique; Evidence quality: intermediate; Strength of recommendation: strong). If a patient in the maximal setting had an incomplete colonoscopy, a repeat colonoscopy, or if colonoscopy is not feasible, the next tier would be one of the two following: CT colonography, if the local radiology service can demonstrate competency in this technique, or barium enema (if nothing else is possible). If a patient in the limited setting had an incomplete colonoscopy, barium enema may be offered (Note: colonoscopy may not have been available; therefore incomplete colonoscopy would not apply.) (Type: informal consensus; Evidence quality: insufficient; Strength of recommendation: strong).

#### Source guidelines and discussion.

These recommendations are based on NICE and CCO guidelines when clinicians (likely primary care providers) see patients with suspected cancer. In all settings, a complete physical examination that includes a DRE should be performed. DRE is a simple maneuver that can provide valuable information if a suspicious rectal mass is palpated and lead to expedited referral. This is supported by prospective studies (the CCO guideline cited some examples) showing a positive predictive value for DRE, in the presence of other symptoms, of greater than 5%. The optimal strategy for the workup and diagnosis of patients with suspected colorectal cancer without contraindications to colonoscopy is referral for optical colonoscopy. The Expert Panel recommends colonoscopy or flexible sigmoidoscopy with biopsy (if contraindications to colonoscopy) if referral to those resources/services is available. However, if neither is available in resource-constrained settings and/or if the clinical team determines that a colonoscopy cannot be performed in a clinically reasonable time, either locally or by referral, the Expert Panel consensus is to recommend barium enema, if available, after a complete physical examination that includes DRE.

Although this ASCO guideline Expert Panel did not conduct a systematic review comparing interventions, the miss rate for double contrast barium enema, in one study from the United Kingdom noted by a panel member, was 26.7%.^21^ The Expert Panel acknowledges that a barium enema can miss approximately this percentage of polyps. However, in basic or limited settings where access to colonoscopy is not possible or there is an excessive wait time (more than 4 to 6 weeks), the next best strategy in patients with rectal bleeding may be barium enema. (Barium enema is a choice in the NICE 2015 guideline^22^; the wait time issues are supported by the CCO guideline^11^). Patients who have positive findings that increase suspicion for a colonic mass or polyp on a barium enema should be referred to surgery. The NICE diagnostics guidance recommends testing for fecal occult blood for patients without rectal bleeding but with unexplained symptoms who do not meet the criteria for a suspected colon cancer care pathway (Note that ASCO did not formally review this, as it was not in the scope of the original search for guidelines).^23^ The CCO guideline (its 2017 guideline) does not recommend this.^11^

For patients with contraindications to colonoscopy (or when colonoscopy is not available) but without contraindications to flexible sigmoidoscopy, an unsedated flexible sigmoidoscopy is reasonable. However, the quality of sigmoidoscopy and depth of insertion of the sigmoidoscope in actual practice are commonly suboptimal. In a study suggested by a panel member but outside of the ASCO search parameters, examination of the entire sigmoid was not achieved in approximately a quarter of patients, and the descending colon was intubated only in a minority of cases.^24^ Another limitation of sigmoidoscopy is that more than a third of colon cancers are located proximal to the splenic flexure. Another suggested study (also outside of the inclusion criteria) demonstrated a significant proximal shift in colon cancer distribution over time, with 36.7% of cancers located proximal to the splenic flexure.^25^ Flexible sigmoidoscopy may be indicated for certain patients, but it should not replace colonoscopy as the first-choice investigation in patients with symptoms suspected of having colorectal cancer if colonoscopy is available and the patient does not have contraindications to receiving it.

There are very few contraindications to flexible sigmoidoscopy or unsedated colonoscopy (preferably done with water immersion). Patients with severe or advanced cardiopulmonary disease who are not good candidates for surgery, or those with other rare contraindications in enhanced/maximal settings, should/may be referred for FOBT (preferably FIT) and for a CT scan, if available. CT colonography has been shown to be an effective modality in the screening of colon cancer but has not been thoroughly evaluated in patients who are symptomatic and suspected of having colorectal cancer. Thus, CT colonography is not an optimal strategy but might be an alternative for this specific scenario. Conventional CT scan (not requiring an oral purge) is easier and may be preferred if colonoscopy is not available. If CT is not available either locally or by referral in basic or limited settings, then a barium enema is recommended. Given that patients with contraindication for colonoscopy or sigmoidoscopy will be unable to undergo tissue diagnosis, CT imaging may be appropriate, if available, for follow-up of abnormal results.

Based on this discussion, optical colonoscopy is the dominant strategy for the evaluation of disease in symptomatic patients. Basic or limited settings may have restricted access to colonoscopy, but policymakers should plan to allocate resources to make that an available option for accurate diagnosis.

In patients who have an incomplete or limited colonoscopy, the best strategy is to repeat colonoscopy under optimal conditions, including high-quality bowel preparation, adequate sedation, and referral to expert endoscopy centers. If that is not possible, then CT colonography is suggested; if that is not possible, then barium enema is suggested.

## COST IMPLICATIONS

A literature search focusing on high-quality systematic reviews of published cost-effectiveness analyses on low-resource settings was conducted, and none were found. The Expert Panel therefore identifies the need for cost-effectiveness analyses of early detection from low-resource settings. A table of individual cost-effectiveness analysis studies is found in the Data Supplement.

## EXTERNAL REVIEW AND OPEN COMMENT

The draft recommendations were released to the public for open comment from July 13 through July 27, 2018. Response categories of “Agree as written,” “Agree with suggested modifications,” and “Disagree. See comments” were captured for every proposed recommendation with two written comments received. The two respondents either agreed or agreed with slight modifications with 100% of the recommendations and disagreed with no recommendations. Expert Panel members reviewed comments from all sources and determined whether to maintain original draft recommendations, revise with minor language changes, or consider major recommendation revisions. All changes were incorporated before CPGC review and approval.

The draft was submitted to one external reviewer with content expertise. The draft quality was rated as high, and it was agreed that the guideline would be useful in practice. Review comments were reviewed by the Expert Panel and integrated into the final manuscript before approval by the CPGC.

## LIMITATIONS OF THE RESEARCH AND FUTURE DIRECTIONS

There were limitations on the evidence to inform some of the recommendations. Limitations on published data include the following:

- Most studies cited in the maximal setting guidelines that this guideline adapted were conducted with populations in high-resource settings.
- There is a lack of direct head-to-head trials of screening modalities and in staging modalities (latter per NICE guideline^15^).
- There is a lack of data on age ranges in low-resource settings for screening.
- There is a lack of data on intervals in low-resource settings for screening.
- According to empiric data about the effectiveness of different screening strategies for blacks and Alaska Natives (USPSTF) and optimal age ranges in these non-white US groups.^9^
- There is a lack of high-quality data on the management of patients with polyps.
- There is a lack of data on preprocedural management of polypectomy setting.
- There is a lack of data on management of disease in patients receiving new anticoagulation agents (eg, rivaroxaban and dabigatran) who will undergo polypectomies for sessile lesions.
- Use of tattoo or alternative for marking for surgical resection guidance needs more evidence.

### Suggestions for Future Research

- Head-to-head trials of screening modalities
- Understand the endoscopy capacity of various settings, including basic to enhanced-resource settings, to determine within each region/country the capacity for evaluation of positive screening test. This understanding should include facilities, expertise, and equipment. Surveys of endoscopy capacity are warranted for assessment of resources.
- Cost-effectiveness research and modeling of screening versus treatment modalities
- Assess the impact of low-cost, single FIT or FOBT use in previously nonscreened populations.
- Evaluate quality for complex polyp management, including presence of multidisciplinary boards, endoscopist experience, and additional polyp characteristics to predict unresectability.
- Screening trials in racial and ethnic minority groups

The Expert Panel suggests that research should be conducted on these topics.

ASCO believes that cancer and cancer prevention clinical trials are vital to inform medical decisions and improve cancer care. All patients should have the opportunity to participate.

## ADDITIONAL RESOURCES

Additional information, including data supplements, evidence tables, and clinical tools and resources, can be found at www.asco.org/resource-stratified-guidelines. Patient information is available there and at www.cancer.net.

**Related ASCO Guidelines**

- Palliative Care in the Global Setting (http://ascopubs.org/doi/10.1200/JGO.18.00026)^26^
- Patient-Clinician Communication (http://ascopubs.org/doi/10.1200/JCO.2017.75.2311)^27^
- Treatment of Patients With Early-Stage Colorectal Cancer (http://ascopubs.org/doi/10.1200/JGO.18.00214)^28^

## Figures and Tables

**TABLE A1:**
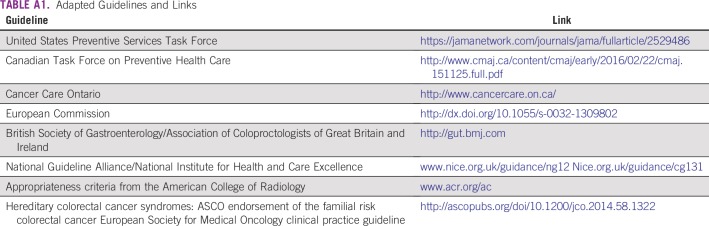
Adapted Guidelines and Links

**TABLE A2:**
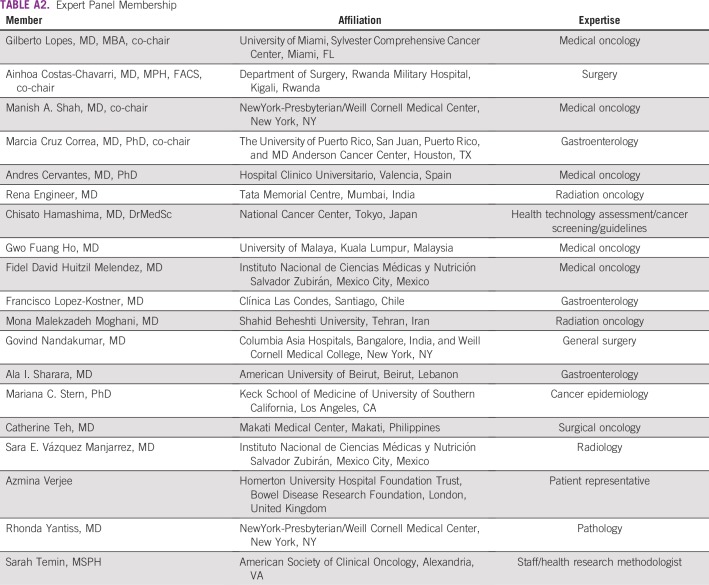
Expert Panel Membership
